# Advancing rapid adaptation for urgent public health crises: Using implementation science to facilitate effective and efficient responses

**DOI:** 10.3389/fpubh.2022.959567

**Published:** 2022-08-24

**Authors:** Andria B. Eisman, Bo Kim, Ramzi G. Salloum, Clayton J. Shuman, Russell E. Glasgow

**Affiliations:** ^1^Community Health, Division of Kinesiology, Health and Sport Studies, College of Education, Wayne State University, Detroit, MI, United States; ^2^Center for Health and Community Impact (CHCI), Wayne State University, Detroit, MI, United States; ^3^Center for Healthcare Organization and Implementation Research, VA Boston Healthcare System, Boston, MA, United States; ^4^Department of Psychiatry, Harvard Medical School, Boston, MA, United States; ^5^Department of Health Outcomes and Biomedical Informatics, University of Florida College of Medicine, Gainesville, FL, United States; ^6^Department of Systems, Populations, and Leadership, University of Michigan School of Nursing, Ann Arbor, MI, United States; ^7^Dissemination and Implementation Science Program of ACCORDS (Adult and Child Consortium for Health Outcomes Research and Delivery Science), University of Colorado School of Medicine, Aurora, CO, United States

**Keywords:** implementation science, health services research, decision making, emergencies, economics, economic models, rapid cycle, COVID-19

## Abstract

Responding rapidly to emerging public health crises is vital to reducing their escalation, spread, and impact on population health. These responses, however, are challenging and disparate processes for researchers and practitioners. Researchers often develop new interventions that take significant time and resources, with little exportability. In contrast, community-serving systems are often poorly equipped to properly adopt new interventions or adapt existing ones in a data-driven way during crises' onset and escalation. This results in significant delays in deploying evidence-based interventions (EBIs) with notable public health consequences. This prolonged timeline for EBI development and implementation results in significant morbidity and mortality that is costly and preventable. As public health emergencies have demonstrated (e.g., COVID-19 pandemic), the negative consequences often exacerbate existing health disparities. Implementation science has the potential to bridge the extant gap between research and practice, and enhance equity in rapid public health responses, but is underutilized. For the field to have a greater “real-world” impact, it needs to be more rapid, iterative, participatory, and work within the timeframes of community-serving systems. This paper focuses on rapid adaptation as a developing implementation science area to facilitate system responses during public health crises. We highlight frameworks to guide rapid adaptation for optimizing existing EBIs when responding to urgent public health issues. We also explore the economic implications of rapid adaptation. Resource limitations are frequently a central reason for implementation failure; thus, we consider the economic impacts of rapid adaptation. Finally, we provide examples and propose directions for future research and application.

## Introduction

Public health emergencies are health-related events for which the timing, scale, and unpredictability threaten the capability of clinical, community, and public health systems to effectively manage them (1). In response to these urgent issues, a range of treatment and prevention interventions across a variety of settings are utilized to stem their onset and escalation. But *how* interventions are implemented is as important as *what* interventions are implemented (2). Implementation science has significant and underdeveloped potential to facilitate the rapid adaptation of evidence-based interventions (EBIs; which can include programs, practices, and/or policies) to meet population needs and minimize morbidity and mortality in response to a crisis (3).

The COVID-19 pandemic highlighted the critical role of dissemination and implementation (D&I) science in facilitating the rapid and effective uptake of EBIs during urgent or emerging public health events. The D&I of interventions often involves adaptations to existing practices, policies, workflows, and priorities; this is even more challenging under the time- and resource-constraints related to urgent issues (4). The consequences of not incorporating a D&I focus in addressing urgent issues can include delayed services, wasted resources, inequities in service access and delivery, and ultimately poor public health outcomes (4).

Responding to emergent public health issues are challenging and disparate processes for researchers and practitioners. Traditional research-to-practice translation approaches that are both time- and resource-intensive are inadequate to address urgent public health crises (3). Researchers often develop new interventions using the traditional, linear research process that takes years and significant resources, generally with little exportability or external validity once the initial efficacy trials are completed (5). Yet, developing and testing new interventions for each emerging crisis is unlikely to meet population needs, fit health organizations' timeframes, and likely to result in a widening of the research-to-practice gap. Practitioners, health clinics, schools, and other systems, in contrast, are often poorly equipped to identify and adopt EBIs for rapid dissemination during a crisis (2). Research evidence is infrequently communicated in a way that is accessible and pragmatic for practitioners and systems to apply (6). For D&I science to have a greater “real-world” impact it needs to be more rapid, iterative, and work collaboratively within the timeframes and capacities of systems that serve communities (7, 8).

### Health equity in D&I research and rapid adaptation

As the COVID-19 pandemic and other public health emergencies have demonstrated, marginalized and disadvantaged populations often suffer the negative consequences disproportionately (9). During the pandemic, we observed elevated morbidity and mortality and secondary consequences such as exacerbating mental health and substance use issues among marginalized populations who have the least access to treatment and prevention services (9, 10). Researchers suggest that those who could most benefit from EBIs may also be the least likely to receive them as intended -referred to as the “inverse prevention law (11).” This may be especially true with urgent public health issues. Rapid responses are often needed for all populations, but with attention to specific gaps that may contribute to inequities. Health equity may be the “central indicator of success” for implementation research, but this requires a greater explicit focus on meeting the needs of higher-risk populations (11, 12).

In the current paper, our purpose is to advance the application of D&I science to address urgent public health issues through rapid adaptations. We address the following objectives: (1) summarize recent work on rapid adaptations within implementation science, including specific theories and frameworks; (2) provide examples and identify strengths and limitations of this work, and (3) discuss future directions for research and practice on rapid adaptations.

## D&I science and rapid adaptation

**Figure 1:**
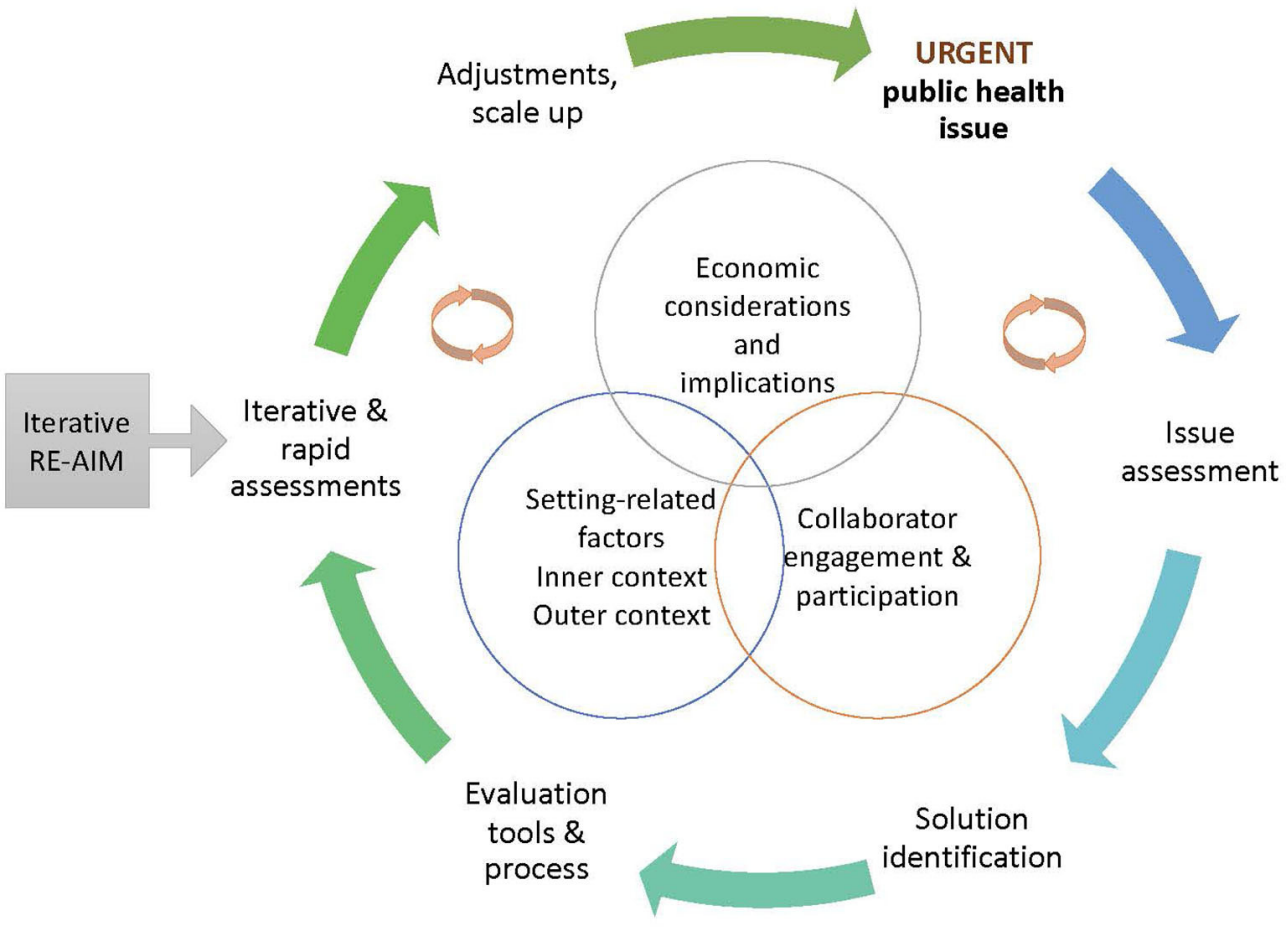
Common steps across the rapid adaptation process: collaborator engagement, setting-related factors, and economic implications are central as are the cyclical, rapid, iterative nature of the processes.

Structured approaches to rapid adaptations, based on conceptual models or research design principles, are needed. Rapid research methods, used to guide rapid adaptations, are not unique to D&I science; fields such as human factors engineering and frameworks such as human-centered design have long embraced rapid-cycle research to iteratively improve (i.e., adapt) products and processes to effectively and efficiently meet end-user needs (13–16). D&I science is learning from these areas, and while not comprehensive, we highlight several D&I approaches for rapid responses (also see Figure 1). We summarize common steps and demonstrate their application in the case examples and discuss collaborator engagement; setting-related factors; and economic implications as central in the rapid, iterative nature of the process.

### Selected frameworks to inform rapid responses

#### After action review

An After Action Review (AAR) is a learning-driven constructive review of actions taken in preparation for, during, and following a public health event, to inform changes to effectively address impending, ongoing, and future crises (17). AARs have been successfully used by the World Health Organization (WHO) and others to systematically learn from both emergent events [e.g., natural disasters; (18, 19)] and planned events [e.g., quality improvement initiatives; (20, 21)]. AAR culminates in an actionable report of (1) what is expected to happen under existing procedures, (2) what is actually happening, (3) what is going well and why, and (4) what can be improved and how. AAR can rapidly identify needed changes to an EBI under crises, primarily through employing collaborator engagement procedures (e.g., constructive, improvement-focused facilitated discussions) that efficiently and systematically gather multiple perspectives to assess the issue, identify solutions, review their impact, and make further adjustments, as depicted in Figure 1. Specifically, AAR leads to methodical delineation of (i) current planned tasks for EBI delivery, (ii) tasks actually being conducted, (iii) planned tasks achieving stated objectives to retain as-is, and (iv) tasks that can be improved *via* rapid adaptations that allow effective intervention delivery under crises.

#### Rapid cycle research

Rapid Cycle Research (RCR), according to the Agency for Healthcare Research and Quality (AHRQ), is a process in which urgent problems are identified and addressed using incremental, contextually informed methods [see steps and context, Figure 1; (22)]. As defined at a recent meeting sponsored by the National Cancer Institute building on this initial work (23), rapid-cycle research (RCR) has six characteristics: iterative design; proximal outcome focus; partner engagement emphasis; setting and context focus; consider data sources, and incorporate appropriate rigor. While some of these characteristics are found in other research areas, their *combination* constitutes RCR. RCR studies, importantly, may place greater or lesser emphasis on each of these characteristics. The rigor characteristic is intended to address potential objections that rapid research necessarily sacrifices rigor or methodology standards. Although not listed as a key characteristic, RCR also focuses on the efficiency of research, that is maximizing outcomes while minimizing resource use (see economic implications section).

#### Iterative RE-AIM

The RE-AIM (Reach Effectiveness Adoption Implementation and Maintenance) framework has been widely used to evaluate implementation and plan programs. Iterative RE-AIM provides a conceptually and data-based approach to rapid research (24). In Iterative RE-AIM, periodically throughout implementation partners and implementation teams assess progress on the various RE-AIM outcome dimensions and current priorities across these dimensions (see Figure 1). In the Iterative component, team members determine current priorities and the gap between priority and progress on each RE-AIM dimension. They then collaboratively discuss outcome targets and decide upon adaptations for the next time period.

## Economic implications of D&I science for rapid adaptation

Implementing EBIs in response to crises has numerous and immediate economic implications. Economic evaluation methods used to assess costs and outcomes of public health systems interventions (25) can also inform whether rapid adaptations represent a cost-effective use of limited resources. Important costs related to rapid responses include intervention costs, implementation costs, including implementation strategies, and downstream costs (26). Implementation strategy costs to engage in rapid adaptation (solution identification, Figure 1) may include resources for intervention tailoring (i.e., promote adaptability), conducting ongoing training, developing an implementation blueprint, and resources dedicated to ongoing quality monitoring of the intervention to ensure its safety and effectiveness (evaluation tools, iterative assessments, Figure 1), and potentially downstream costs.

Whether rapid implementation strategies are making health organizations more efficient is another key consideration. Efficiency may include, for example, technical efficiency which measures the quantity of outputs produced relative to inputs used. Allocative efficiency examines whether an organization uses inputs, given their prices, in a way that minimizes total costs (27). Relative efficiency considers the diversity of inputs and outputs used in healthcare and the range of implicit valuation on various intervention components placed by organizations and systems. Prospective economic modeling can inform health practitioners and policymakers planning to deploy strategies for rapid adaptation and scale-up EBIs by projecting the expected value and impacts of various levels of efficiency.

### Case examples

#### AAR to rapidly adapt residential treatment programs' responses to COVID-19

We applied the AAR framework to learn from residential treatment programs' COVID-19 responses and identify changes to inform subsequent waves of the pandemic (28). We examined two Mental Health Residential Rehabilitation and Treatment Programs of the United States Department of Veterans Affairs [VA; (29)]. These programs have around-the-clock staffing for residents in need of behavioral health care and/or experiencing homelessness. The AAR included five phases (Design, Prepare, Conduct, Debrief, and Follow-up) conducted over seven months, approximately 2 months of which involved engaging program personnel in improvement-focused discussions and rapidly identifying context-specific adaptations. The other preparatory and follow-up months were for initially establishing AAR procedures and iteratively pursuing continuous improvement, respectively, which can be expedited for future AAR applications and undertaken in parallel with operationalizing identified adaptations.

For the Design phase, we devised involving program staff and residents to conduct small-group virtual discussions of four to seven individuals per group. For the Prepare phase, our semi-structured guide included: What operating procedures were established/revised for COVID-19? What cooperation with other organizations occurred? What physical/mental health issues arose more/less frequently since COVID-19? What policies worked well or need revision? For the Conduct phase, we incorporated additional probing questions aligned with the AAR framework: What was planned? What actually happened? What went well and why? What can be improved and how? For the Debrief phase, we summarized and shared our findings with program and health care system leadership. Recommended adaptations identified included (i) conveying reasons for COVID-related precautions/changes to residents, (ii) keeping COVID-related information sharing and recovery-oriented programming separate, (iii) providing “how to use technology” training during program orientation, and (iv) developing procedures for safe family interactions and off-site activities. For the Follow-up phase, rapid adaptations identified in the AAR discussions included (i) providing details for COVID-related precautions/change during all-resident community meetings, (ii) de-emphasizing COVID-related information during treatment groups, (iii) consolidating all remote programming under one technology platform, and (iv) when COVID prevalence is low, granting family visit passes.

#### Rapid adaptation of a physical activity intervention during COVID-19

InPACT (Interrupting Prolonged sitting with ACTivity) is a school-based intervention for children and youth focused on increasing physical activity levels with short bursts of structured activity breaks throughout the day (30). InPACT was originally developed based on principles of designing for dissemination to support implementing core functions (e.g., PA: physically active time) and permit flexibility to meet the unique needs and resources of the setting; InPACT includes a compendium of implementation strategies to support flexibility and uptake (31, 32).

The COVID-19 pandemic posed new challenges to youth PA, with low-resource communities disproportionately impacted (33, 34). The widespread shutdowns, including schools, imposed significant barriers to exercise opportunities; consequently, PA among youth plummeted, especially among racial/ethnic minorities in low-resource environments (35). The Vice President of Michigan's Board of Education created a PA dissemination task force and chose InPACT to rapidly adapt and disseminate to mitigate this urgent public health issue. The 3-month process of rapidly adapting InPACT from school to the home was guided by Rapid-Cycle Research and Iterative RE-AIM. The details of the adaptation process are described elsewhere (36), but are briefly described here.

The steps included: Step 1: identifying partner organizations aligned with the goal of improving PA; this included PE teachers, state agencies, professional organizations (e.g., principals' association), school health coordinators, and professional sports teams. Steps 2 and 3: engaging in problem and knowledge exploration. The research team hosted community forms with parents, teachers, administrators, and community members to aid in understanding the scope of the problem. Steps 4 and 5: initiating solution development and testing. The task force identified InPACT as the simplest and most scalable solution. The intervention development team adapted InPACT for home delivery (e.g., creating asynchronous PA videos) and used iterative RE-AIM to assess progress. Partner organizations/teams met periodically throughout the implementation process and assessed progress on their identified RE-AIM outcome dimensions given each team's priorities and objectives. For example, the InPACT development team assessed Reach by the number of partner organizations that were actively disseminating InPACT through various channels (e.g., public TV). Iterative RE-AIM offered each team member the opportunity to capitalize on their strengths and make adjustments based on the most important dimensions of their efforts. Step 6: utilizing coordinated, active dissemination strategies across collaborators (e.g., press releases, promotion through partner organizations) to enhance the reach of InPACT@home.

#### Limitations of implementation science and rapid adaptation

Although rapid adaptation may be essential for responding to emerging public health crises, it also has the potential to create adverse sequelae and unintended consequences. In response to surging COVID-19 cases, hospitals around the world rapidly revised and implemented new visitation policies (37–39). The swift enactment of adapted visitation policies significantly affected patients and their family members. For example, in maternal-infant health settings, many of these rapidly adapted policies restricted support persons (e.g., partners or doulas) during labor (40), and, specifically in neonatal intensive care settings, the separation of mothers and/or fathers from their infants (41). Emerging literature highlights the negative consequences of these adapted infection prevention policies on patient outcomes (39).

The example of rapidly adapted policies highlights two critical points as we consider advancing rapid adaptation in D&I science: The need to (1) identify and assess the potential for immediate and long-term adverse effects before engaging in rapid adaptation and (2) prepare to address the occurrence and magnitude of potential downstream adverse effects. Additionally, iterative and rapid assessments, as with iterative RE-AIM or other audit and feedback processes (see Figure 1), are vital so practices or policies can be modified or abandoned if proven harmful (4). Involving community partners in planning for and guiding rapid adaptations (see collaborator engagement, Figure 1), and curating available resources and supports should reduce the likelihood and/or magnitude of adverse or subsequent effects resulting from rapid adaptation.

#### Creating synergy: D&I science and related fields

There is increasing dialogue and synergy between D&I science and related fields that can especially be leveraged to fuel rapid adaptation-related developments. The first is with improvement science. These fields share purpose, scope, and methods and are at similar stages of scientific discipline development (42, 43). This synergy is expected to grow as D&I's focus on improving EBI implementation extends to demonstrating successful impact on care quality, value, and safety (i.e., foci of improvement science) and as improvement science integrates principles of both implementation science and quality improvement. The synergy can provide fertile ground for adopting well-established iterative quality improvement approaches and concepts for use by implementation science for rapid adaptations. The second area is human-centered design, which focuses on shaping products (e.g., EBIs) to be grounded in the people and settings who will use the innovation (44). Efforts to apply human-centered design approaches for EBI implementation (45–47) emphasize iteratively updated contextual needs assessments and updated interventions *and* implementation strategies. As implementation science continues to draw on human-centered design, the latter's in-depth and constant focus on user needs, prototype testing, and contextual alignment can undoubtedly aid with rapid adaptations that enable better implementation of EBIs into target settings.

## Discussion

Several cross-cutting issues emerge from the rapid adaptation approaches and examples discussed (see Table 1):

**Table 1:** Cross-cutting issues in rapid adaptation.

**Cross-cutting issue**	**Description**	**Recommendation**
Suitability of rapid adaptation approach	Opportunity costs and costs of being wrong vs. inaction	Assess if the potential benefits of rapid adaptation outweigh the risks
Pragmatic data sources and evaluation Prioritize what to assess Check and vet data	Are there reliable, rapid responsive data on which to make decisions	Start with participation and equity-related data e.g., adoption and reach, collaborator voice in which aspects to evaluate
Collaborator engagement	Need teams that have established trust and working relationships	Form a response team of multi-sector collaborators before the crisis; use different collaborators for different purposes
Reduce risks/optimize benefits	Anticipate possible risks and benefits of rapid adaptation	Conduct ongoing iterative assessments, use simulation modeling
Allocating and leveraging resources	Consider context-specific resources and those allocated and available, opportunity cost	Develop a preliminary plan for crises; identify and describe adaptations to programs and resources that can be made rapidly as needed in response to crises and disasters; assess resource use iteratively, making adjustments
Equity impacts	Potential for unintentionally exacerbating inequities	Vet strategies with those to be impacted Have rapid data systems on equity impacts

(1) The suitability of a rapid adaptation application: Not all issues lend themselves to rapid adaptation. For example, if the costs of being wrong are substantial or if the only data available are significantly lagging behind implementation, this may not be the best approach. (2) Pragmatic data sources and evaluation: There are recent examples of developing valid, close-to real-time data (48). However, obtaining and checking data for accuracy can itself be time-consuming. Organizations and systems may need to create an infrastructure, for example, to curate reliable electronic health record (EHR) data (49). Given truncated timeframes, prioritizing data that are central to decision-making is also important when considering data sources and evaluation. (3) Collaborator engagement and “going slow to go fast.” Developing trust and working across different partners takes time, but once these relationships are established, research can proceed more quickly. (4) Allocating and leveraging resources: The need to leverage existing resources includes creating ways to deliver programs and evaluations using available resources and staff, or routinely collected secondary data. A less frequently recognized rapid adaptation need is relying on institutional memory, especially how similar EBIs adaptations have worked in the past. (5) Reduce risks and optimize benefits: Another cross-cutting issue is the need to prevent and address potential risks to rapid adaptations. Unintended consequences, especially adversely impacting health and equity, need to be considered. Such outcomes can be mitigated by strategies including partner engagement that ensures representation from impacted groups, rapid participatory modeling-including costs and benefits, and including measures of health disparity as key outcomes. Rapid iteration along with continual evaluation may be the single best way to address potential harms as well as other challenges such as limited experimental rigor. (6) Equity impacts: Although sample sizes are often small in rapid research, settings and participants can be purposefully selected to include diversity and characteristics especially important for generalization (e.g., including low-resource settings that have high staff turnover; participants with social health challenges or having experienced health inequities). Ensuring that given adaptations achieve equitable impact will support a greater focus on how to meet the needs of populations at high risk of experiencing the negative impacts of public health events (12).

As argued by Chambers and Norton, adaptations to the EBI itself may be required to better fit the context in which implementation occurs but must retain its core elements to achieve the intended benefit (50). Similarly, implementation strategies may also need modification to suit the context and in response to evolving challenges (51, 52). Moreover, to effectively address a crisis, several different EBIs and implementation strategies may warrant being employed simultaneously, and this, too, may require tailoring to the context. For example, there are multiple COVID-19 prevention measures including vaccines, masks, and physical distancing, and how they are relatively prioritized and implemented alongside one another may need to be modified based on cultural norms, available resources, and other contextual characteristics of different settings.

As adaptations are central to implementation, Wiltsey Stirman et al. (53) developed the Framework for Reporting Adaptations and Modifications to Evidence-based interventions (FRAME) to advance adaptation measurement. Although FRAME was not developed exclusively for rapid adaptations, elements of it and the companion FRAME-IS for evaluating adaptations to implementation strategies (54) can be applied to rapid research. Reporting and measuring modifications to EBIs and implementation strategies is critically important, and examples, recommendations, and frameworks are available (53, 55).

The COVID-19 pandemic has underscored the urgency of advancing D&I science to guide effective, rigorous, and efficient rapid adaptations. We conclude that making science more rapid is vital to reducing morbidity and mortality during public health crises. We acknowledge that the relative contribution and costs of rapid adaptations need to be carefully considered and monitored to ensure they achieve desired objectives. In this paper, we provide preliminary guidance on rapid adaptation based on data and theory from D&I and related disciplines. As an emerging area in D&I science, rapid adaptation has notable potential to support conceptual and data-driven decision-making during crises to minimize negative public health impacts.

## Data availability statement

The original contributions presented in the study are included in the article/supplementary material, further inquiries can be directed to the corresponding author/s.

## Author contributions

All authors listed have made a substantial, direct, and intellectual contribution to the work and approved it for publication.

## Funding

Preparation for this manuscript was in part supported by K01DA044279, PI: Eisman, National Institute on Drug Abuse, National Institutes of Health. Publication fees are supported by Wayne State University.

## Conflict of interest

The authors declare that the research was conducted in the absence of any commercial or financial relationships that could be construed as a potential conflict of interest.

## Publisher's note

All claims expressed in this article are solely those of the authors and do not necessarily represent those of their affiliated organizations, or those of the publisher, the editors and the reviewers. Any product that may be evaluated in this article, or claim that may be made by its manufacturer, is not guaranteed or endorsed by the publisher.
